# Serum retinol binding protein 4 is negatively related to beta cell function in Chinese women with non-alcoholic fatty liver disease: a cross-sectional study

**DOI:** 10.1186/1476-511X-12-157

**Published:** 2013-10-27

**Authors:** Hongmei Yan, Xinxia Chang, Mingfeng Xia, Hua Bian, Linshan Zhang, Huandong Lin, Gang Chen, Mengsu Zeng, Xin Gao

**Affiliations:** 1 Department of Endocrinology and Metabolism, Zhongshan Hospital, Fudan University, 180 Fenglin Road, Shanghai 200032, China; 2 Department of Radiology, Zhongshan Hospital, Fudan University, Shanghai 200032, China

**Keywords:** Serum retinol binding protein 4 (RBP4), Beta cell function, Non-alcoholic fatty liver disease (NAFLD)

## Abstract

**Background:**

To observe the relationship between serum retinol binding protein 4(RBP4) and β cell function in Chinese subjects with non-alcoholic fatty liver disease (NAFLD) and without known diabetes.

**Methods:**

106 patients diagnosed as fatty liver by ultrasonography (M/F: 61/45; aged 47.44 ± 14.16 years) were enrolled in our current cross-sectional study. Subjects with known diabetes, chronic virus hepatitis and excessive alcohol consumption were excluded. Serum RBP4 was detected by ELISA and validated by quantitative Western blotting. β cell function were assessed by HOMA in all subjects and by hyperglycemic clamp in 17 normal glucose tolerance subjects (M = 6, F = 11).

**Results:**

The levels of serum RBP4 in men were higher than that in women (55.96 ± 11.14 vs 45.87 ± 10.31 μg/ml, p < 0.001). Pearson’s correlation analysis demonstrated that in women, serum RBP4 levels were significantly associated with fasting blood glucose (FBG), HOMA-β, and increment of first phase insulin secretion (1PH), but not associated with age, BMI, waist circumference, WHR, systolic (SBP) and diastolic blood pressure (DBP), TC, TG, HDL-c, LDL-c, 2 h blood glucose, HOMA-IR, ALT, AST, γ-GT, hepatic fat content (HFC), and insulin sensitivity index (ISI). However, in men, serum RBP4 levels were significantly associated with HDL-c, ALT, AST, but not associated with any other parameters as mentioned above. A stepwise multiple linear regression analysis demonstrated that in women, HOMA-IR and RBP4 were significantly associated with HOMA-β, while in men, HOMA-IR and BMI were significantly variables associated with HOMA-β.

**Conclusions:**

Serum RBP4, secreted mainly by liver and adipose tissue, may involve in the pathogenesis of β cell dysfunction in Chinese women patients with NAFLD.

## Background

Retinol binding protein 4 (RBP4) is a member of the lipocalin family of proteins that transport retinol from the liver to the peripheral tissues and plasma RBP4 levels positively correlate with retinol levels.

A potential link between RBP4 and type 2 diabetes mellitus was suggested by Yang et al. [1]. The study reported that RBP4 was elevated in insulin-resistant mice with adipose tissue-specific GLUT4 knockout and humans with obesity and type 2 diabetes mellitus. They found that RBP4 expression was increased in insulin resistant adipose tissue specific GLUT4 null mice and reduced in insulin sensitive adipose specific GLUT4 transgenics mice. Transgenic overexpression of human RBP4 or injection of recombinant RBP4 in normal mice causes insulin resistance and glucose intolerance. Conversely, genetic deletion of Rbp4 enhances insulin sensitivity.

Recent studies [2,3] in human found that plasma RBP4 levels were elevated in subjects with IGT or type 2 diabetes mellitus and that RBP4 was related to various clinical parameters known to be associated with insulin resistance. And a cross-sectional study conducted in Chinese found that increased RBP4 levels increased the risk for hyperglycemia, including impaired glucose regulation and newly diagnosed type 2 diabetes mellitus, even after adjustment for a number of confounders [4]. These studies suggested that RBP4 was an adverse impact factor of diabetes mellitus.

Some researchers explored the relationship of RBP4 and insulin resistance. It was reported that serum RBP4 levels associated negatively with insulin sensitivity (determined by a hyperinsulinemic euglycemic clamp) in non-diabetic [5] or impaired glucose metabolism participants [6] and in women with polycystic ovary syndrome [7] or in women with normal glucose tolerance with different obesity [8]. However, the associations between RBP4 levels and insulin resistance were not consistent. Several studies failed to find a significant correlation between RBP4 levels and insulin resistance [9,10]. The suggested explanations for these discrepancies include the differences in renal clearance of RBP4 caused by the different renal functions in the study subjects, the imbalance between RBP4 and retinol, the influence of the collection method, and the antibodies used in measuring RBP4 [11].

Since the relationship between RBP4 and insulin resistance was controversial, and impaired β-cell was another important pathogenesis of diabetes mellitus, the relationship between β cell function and RBP4 need to be further studied. However, few studies showed their relation at present.

Our earlier work found that, the first phase or early phase of insulin secretion were impaired in non-alcoholic fatty liver disease (NAFLD) patients when their hepatic fat content detected by ^1^H MRS or CT were more than 10%, which indicated that liver cell with excessive fat deposition may release some cytokines to affect the beta cell function [12,13]. Recent studies found that levels of serum RBP4 were increased in NAFLD patients [14], so we speculated that the serum RBP4 may be one of cytokines involved in the cross-talk of adipose tissue and beta cell function in patients with fatty liver.

Thus, the purpose of the present study was to investigate correlations between serum RBP4 levels and β-cell function in Chinese NAFLD subjects without known T2DM.

## Results

The clinical characteristics of the subjects are shown in Tables 1 and 2. The women participants were older than men participants. Compared to women, men had higher waist circumference and waist-to-hip ratio (WHR), triglycerides (TG), alanine aminotransferase (ALT), γ-glutamyltranspeptidase (γ-GT), serum RBP4, hepatic fat content, and lower high-density lipoprotein cholesterol (HDL-c). There are no differences between men and women in body mass index (BMI), systolic and diastolic blood pressure, total cholesterol (TC), low-density lipoprotein cholesterol (LDL-c), fasting blood glucose (FBG) and 2 hour blood glucose (2hBG), fasting insulin (FINS) and 2 hour insulin (2hINS), HOMA-IR, HOMA-β, the insulin sensitivity index (ISI), First-phase insulin secretion (1PH), Increment of 1PH, Second-phase insulin secretion (2PH), Increment of 2PH, aspartate aminotransferase (AST), alkaline phosphatase (ALP).

**Table 1:** Basic characteristics of all the subjects

	**Total (n = 106)**	**Men (n = 61)**	**Women (n = 45)**	***P*** **value**
Age (y)	47.39 ± 13.88	44.70 ± 15.21	50.98 ± 11.04	0.016
BMI (kg/m^2^)	26.69 ± 3.48	26.90 ± 3.45	26.39 ± 3.54	0.459
Waist (cm)	91.88 ± 10.04	94.26 ± 9.59	88.64 ± 9.82	0.004
WHR	0.92 ± 0.07	0.95 ± 0.06	0.89 ± 0.07	<0.001
Systolic blood pressure (mmHg)	129.05 ± 19.57	130.67 ± 16.57	126.84 ± 23.04	0.322
Diastolic blood pressure (mmHg)	81.45 ± 11.25	82.93 ± 10.50	79.44 ± 12.02	0.115
Total cholesterol (mmol/L)	4.97 ± 0.83	4.93 ± 0.77	5.03 ± 0.92	0.532
Triglycerides (mmol/L)^lg^	2.15 ± 1.22	2.54 ± 1.37	1.63 ± 0.74	<0.001
HDL-c (mmol/L)^lg^	1.16 ± 0.26	1.06 ± 0.18	1.30 ± 0.27	<0.001
LDL-c (mmol/L)	2.89 ± 0.69	2.81 ± 0.62	2.98 ± 0.76	0.217
Fasting blood glucose (mmol/L)^lg^	5.33 ± 0.76	5.31 ± 0.80	5.35 ± 0.72	0.832
2 h blood glucose (mmol/L)^lg^	7.30 ± 2.52	7.08 ± 2.51	7.62 ± 2.53	0.289
Fasting insulin (mU/L)^lg^	10.85 ± 6.95	10.29 ± 7.01	11.61 ± 6.89	0.354
2 h insulin (mU/L)^lg^	80.51 ± 61.67	77.54 ± 66.68	84.74 ± 54.30	0.574
HOMA-IR^lg^	2.62 ± 1.76	2.46 ± 1.81	2.84 ± 1.70	0.289
HOMA-β^lg^	138.36 ± 110.55	136.84 ± 122.41	140.42 ± 93.48	0.874
ALT (U/L)^lg^	47.23 ± 43.10	56.64 ± 47.86	34.82 ± 32.42	0.011
AST (U/L)^lg^	33.12 ± 20.13	34.51 ± 19.96	31.24 ± 20.45	0.427
ALP (U/L)	73.06 ± 20.76	74.87 ± 21.86	70.68 ± 19.22	0.333
γ-GT (U/L)^lg^	49.79 ± 48.67	60.62 ± 58.24	35.54 ± 26.57	0.006
Serum RBP4 (μg/ml)	51.65 ± 11.85	55.96 ± 11.14	45.87 ± 10.31	<0.001
Hepatic fat content (%)^lg^	7.90 ± 6.89	9.10 ± 6.85	6.26 ± 6.66	0.035

Data are means ± S.D. BMI: body-mass index, WHR: waist-to-hip ratio, HDL-c: high-density lipoprotein cholesterol, LDL-c: low-density lipoprotein cholesterol, HOMA-IR: homeostatic model assessment of insulin resistance, HOMA-β:homeostatic model assessment of β cell function, ALT: alanine aminotransferase, AST: aspartate aminotransferase, γ-GT: γ-glutamyltransferase, RBP4: retinol binding protein 4.

lg: logarithmic transformation with 10 as its base of skewed variables.

**Table 2:** Basic characteristics of NGT subjects underwent hyperglycemic clamp

	**Total (n = 17)**	**Men (n = 6)**	**Women (n = 11)**	***P*** **value**
Insulin sensitivity index (ISI)	15.48 ± 14.41	13.55 ± 15.50	16.53 ± 14.45	0.698
First-phase insulin secretion (1PH)	288.32 ± 169.41	304.60 ± 161.52	279.45 ± 180.64	0.780
Increment of 1PH	77.45 ± 41.96	85.57 ± 33.54	73.01 ± 46.83	0.572
Second-phase insulin secretion (2PH)	120.33 ± 67.90	110.21 ± 50.41	125.84 ± 77.53	0.665
Increment of 2PH	207.75 ± 145.92	216.91 ± 104.04	202.76 ± 169.05	0.856

In all the subjects, serum RBP4 levels were significantly associated with sex, waist circumference, WHR, SBP, DBP, TG, HDL-c, γ-GT, but not associated with age, BMI, TC, LDL-c, FBG, 2hBG, FINS, 2hINS, HOMA-IR, HOMA-β, ALT, AST, ALP, HFC. ISI, 1PH, Increment of 1PH, 2PH, Increment of 2PH. In women, serum RBP4 levels were significantly associated with FBG, HOMA-β, Increment of 1PH; but not associated with age, BMI, and the other variables mentioned above. While in men, serum RBP4 levels were significantly associated with HDL-c, ALT, AST, but not associated with age, BMI, and the other variables mentioned above, either (Tables 3 and 4).

**Table 3:** Pearson’s correlation analysis of serum RBP4 in all the subjects

	**Total (n = 106)**		**Men (n = 61)**		**Women (n = 45)**	
	***r***	***P***	***r***	***P***	***r***	***P***
Gender	-0.423	0.000				
Age (y)	0.061	0.574	0.114	0.432	0.252	0.126
BMI (kg/m^2^)	0.048	0.653	0.034	0.814	-0.018	0.915
Waist (cm)	0.218	0.040	0.069	0.632	0.121	0.468
WHR	0.343	0.001	0.139	0.332	0.264	0.109
Systolic blood pressure (mmHg)	0.247	0.020	0.138	0.332	0.299	0.068
Diastolic blood pressure (mmHg)	0.241	0.023	0.196	0.167	0.124	0.458
Total cholesterol (mmol/L)	0.095	0.382	0.175	0.228	0.076	0.652
Triglycerides (mmol/L)^lg^	0.330	0.002	0.124	0.398	0.188	0.257
HDL-c (mmol/L)^lg^	-0.392	<0.001	-0.281	0.059	-0.183	0.279
LDL-c (mmol/L)	0.049	0.658	0.108	0.476	0.113	0.504
Fasting blood glucose(mmol/L)^lg^	0.063	0.556	-0.123	0.390	0.322	0.048
2 h blood glucose (mmol/L)^lg^	-0.045	0.676	-0.142	0.322	0.277	0.097
Fasting insulin (mU/L)^lg^	-0.051	0.641	0.164	0.266	-0.271	0.104
2 h insulin (mU/L)^lg^	-0.039	0.729	-0.089	0.546	-0.010	0.954
HOMA-IR^lg^	-0.095	0.387	0.119	0.422	-0.211	0.210
HOMA-β^lg^	-0.101	0.360	0.106	0.472	-0.326	0.049
ALT (U/L)^lg^	0.105	0.335	-0.287	0.048	0.168	0.312
AST (U/L)^lg^	-0.077	0.487	-0.304	0.038	0.055	0.752
ALP (U/L)	0.116	0.310	-0.007	0.962	0.171	0.326
γ-GT (U/L)^lg^	0.239	0.034	0.142	0.357	0.042	0.810
Hepatic fat content (%)^lg^	0.199	0.071	0.048	0.737	0.160	0.339

Data are means ± S.D. BMI: body-mass index, WHR: waist-to-hip ratio, HDL-c: high-density lipoprotein cholesterol, LDL-c: low-density lipoprotein cholesterol, HOMA-IR: homeostatic model assessment of insulin resistance, HOMA-β: homeostatic model assessment of β cell function, ALT: alanine aminotransferase, AST: aspartate aminotransferase, γ-GT: γ-glutamyltransferase, RBP4: retinol binding protein 4.

lg: logarithmic transformation with 10 as its base of skewed variables.

**Table 4:** Pearson’s correlation analysis of serum RBP4 in NGT subjects underwent hyperglycemic clamp

	**Total (n = 17)**		**Men (n = 6)**		**Women (n = 11)**	
	***r***	***P***	***r***	***P***	***r***	***P***
Insulin sensitivity index (ISI)	-0.279	0.295	-0.753	0.142	-0.035	0.919
First-phase insulin secretion (1PH)	-0.307	0.247	0.032	0.960	-0.588	0.057
Increment of 1PH	-0.420	0.105	-0.613	0.272	-0.689	0.019
Second-phase insulin secretion (2PH)	0.008	0.976	0.537	0.350	-0.046	0.894
Increment of 2PH	0.234	0.382	0.530	0.359	0.143	0.674

A stepwise multiple linear regression analysis was performed using HOMA-β as a dependent variable. RBP4 was selected as an independent variable together with age, BMI, HFC, ALT, AST, γ-GT, waist circumference, WHR, SBP, DBP, TC, TG, HDL-c, LDL-c, HOMA-IR in all subjects. We also performed stepwise multiple linear regression analysis within each gender. In all subjects, HOMA-IR (Standardized Coefficients Beta = 0.626, p <0.001) together with age (Standardized Coefficients Beta = -0.245, p =0.013) were determined as significant variables associated with HOMA-β. In women, HOMA-IR (Standardized Coefficients Beta =0.521, p =0.001) and RBP4 (Standardized Coefficients Beta = -0.415, p =0.005) were significant variables associated with HOMA-β. While in men, HOMA-IR (Standardized Coefficients Beta =0.591, p <0.001) and BMI (Standardized Coefficients Beta =0.328, p =0.013) were significant variables associated with HOMA-β (Table 5).

**Table 5:** Multiple linear regression analysis

**Coefficients**						
**Model**		**Unstandardized coefficients**		**Standardized coefficients**	**t**	**Sig.**
		**B**	**Std. error**	**Beta**		
All	HOMA-IR	0.090	0.014	0.626	6.538	0.000
	Age	-0.005	0.002	-0.245	-2.563	0.013
Men	HOMA-IR	0.076	0.016	0.591	4.728	0.000
	BMI	0.026	0.010	0.328	2.625	0.013
Women	HOMA-IR	0.085	0.022	0.521	3.817	0.001
	RBP4	-0.012	0.004	-0.415	-3.039	0.005

A stepwise multiple linear regression analysis was performed using HOMA-β as a dependent variable, RBP4 was selected as an independent variable together with age, BMI, waist circumference, Hepatic fat content, alanine aminotransferase, aspartate aminotransferase, γ-glutamyltransferase, waist-to-hip ratio, Systolic and Diastolic blood pressure, Total cholesterol, Triglycerides, high-density lipoprotein cholesterol, low-density lipoprotein cholesterol, HOMA-IR.

## Discussion

In the current study we found that serum RBP4 levels were inversely associated with β cell function in a cohort of Chinese women NAFLD patients without known diabetes mellitus. To the best of our knowledge, this study is the first showing a quantitative correlation between serum RBP4 levels and beta cell function assessed not only by HOMA-β but also by hyperglycemic clamp in Chinese NAFLD patients. Our study added to the growing evidences that serum RBP4,which secreted mainly by liver and adipose tissue, was a potential adverse impact factor for beta cell function and diabetes mellitus mellitus. In addition, the serum RBP4 might be one of cytokines involved in the cross-talk between adipose tissue and beta cell function in patients with fatty liver.

So far, a total of 3 articles reported the relationship between circulating RBP4 and β cell insulin secretion. Similarly, two studies have found that RBP4 negatively related to beta cell function. But the study population and methods to assess insulin secretion were different. Broch et al. reported that [15] circulating RBP4 was negatively associated with acute insulin response (AIRg) (r = -0.48, P = 0.007), calculated as the insulin area during the first 10 min of the frequently sampled intravenous glucose tolerance test, in 107 Spanish men without diabetes mellitus, especially in obese subjects. In multiple regression analyses to predict insulin secretion, RBP4 emerged as an independent factor that contributed independently to AIRg variance (23%) in obese but not in nonobese subjects. There were no related information about women in this study. Another study investigated 867 non-obese Chinese patients with NGT [16] and showed that serum RBP4 correlated with glucose-stimulated insulin secretion, assessed by ΔI_30_/ΔG_30_ (increment in plasma insulin concentration plasma glucose concentration 30 min after the oral administration of 75 g glucose) and the total area under the curve for insulin over 180 min (AUC-I), in NGT non-visceral obesity subjects, but not in NGT visceral obesity subjects and T2DM patients.

This study and the two studies mentioned above all demonstrated that circulating RBP4 may negatively affect β cell function in different subjects. On the contrary, Stefan et al. found that [17] circulating RBP4 did not correlate with insulin secretion (measured by the IVGTT and OGTT) before or after adjustment for age, sex, and insulin sensitivity in a total of 75 Caucasians without type 2 diabetes mellitus. One of the reasons for this difference may due to different study subjects, another reason may be the excessive adjustment for gender. If the author analyzed the relationship of RBP4 and β cell function in men or women respectively, there might be different results.

It is well known that retinol is pathophysiologically linked to β cell function [18,19]. On the other hand, retinol-binding protein circulates in serum, forming a complex with transthyretin (TTR), a transport protein for thyroxine. An investigation disclosed that TTR constitutes a functional component in pancreatic β cell stimulus-secretion coupling [20]. TTR inhibits the binding of RBP to the receptor [21]. Thus, it is possible that increased serum RBP4 prevents TTR from exerting its β-cell stimulus-secretion effects. In fact, circulating RBP4 is highly bound to TTR in a one-for-one stoichiometric ratio, and there is little or no “free RBP4” in circulation [22]. In a recent study [23] using gel filtration chromatography to analyze the RBP4-TTR complex, it was found that increased serumRBP4 remains bound to TTR in insulin resistant states. Because the affinity of RBP4-TTR binding is very strong [22], the relative stoichiometry and affinity of the two proteins in serum could conceivably influence kinetics of RBP4-antibody binding.

The concentration of serum RBP4 varies by gender, generally lower in women than in men, both among adults and children [2,24,25]. Several previous studies hypothesized that sex hormones may play a role in the gender discrepancy. In support of this hypothesis, RBP4 levels were shown to be different between premenopausal and postmenopausal women [26] and vary according to pubertal status [27]. Although serum RBP4 concentration was higher in men than in women, whereas serum RBP4 didn’t show adverse effect on β cell function of men, which suggested there may be some protective factors, such as testosterone, against RBP4 adverse effect on β cell. McInnes et al. found that androgen receptor (AR) signaling in adipocytes not only protects against high-fat diet induced visceral obesity but also regulates insulin action and glucose homeostasis, independent of adiposity. Androgen deficiency in adipocytes in mice resembles human type 2 diabetes mellitus, with early insulin resistance and evolving insulin deficiency [28]. Palomar et al. found [29] that testosterone, other than progesterone nor estradiol, protects the β cells of men pancreas against STZ-induced apoptosis, which is sex specific and could be mediated through the induction of catalase and Cu/Zn-SOD. It demonstrated that androgens may protect β cell function in men.

The relationship about RBP4 negatively related to beta cell function, restricted to some, but not all of the study population, which suggested that their relationship was complicated and influenced by many other confounding factors and deserved further research.

### Limitations

Firstly, the cross-sectional design of the study represents a limitation, implicating that cause-and-effect relationships cannot be discerned. Secondly, we used CT as a tool to determine the hepatic fat content, and its diagnostic accuracy is inferior to pathological diagnosis by liver biopsy. Thirdly, we did not measure blood glucose and insulin at 30 min, so we could not analyze the relationship of serum RBP4 and early phase of insulin secretion. Finally, due to the limitation of conditions, there were only 17 NGT patients undertook clamps in this study. Hence, correlation analysis of serum RBP4 and the increment of 1PH was underpowered because the sample size was too small. So, the data came from hyperglycemic clamp could just be considered as a clue, the real relationship of RBP4 and 1PH or increment of 1PH needs further verification by large-scale studies.

In summary, our results suggest that serum RBP4 may negatively affect β-cell function in Chinese women NAFLD patients without known diabetes mellitus.

## Subjects, materials and methods

### Subjects

This is a cross-sectional study. A total of 106 subjects of Chinese origin (HanChinese) (61 men and 45 women) diagnosed as fatty liver by ultrasound from the outpatient department of Endocrinology of Zhongshan hospital, Fudan university between Nov. 2005 and Apr. 2006 were recruited for this study.

Subjects with excessive alcohol consumption (≥30 g/d in men and ≥20 g/d in women) were excluded from the study. Subjects were also excluded if they had known diabetes mellitus, hepatitis B or C or other liver diseases, or if they were receiving hypoglycemic or lipid-regulating (statins, fibrates) drugs, or other drugs that may impact glucose and lipid metabolism. Other exclusion factors were diseases affecting the metabolic state or not suitable to participate in this study, for example, hyperthyroidism, hypothyroidism, mental disease, serious disease with dysfunction of heart, liver, kidney, and cancer, etc.

All subjects underwent comprehensive physical examinations and routine biochemical analyses of blood. A 75-g oral glucose tolerance test (OGTT) with a test of 2 time-points (0 and 120 min post glucose loading) was performed. Among all subjects, 59 had normal glucose regulation (NGR), 13 had isolated-impaired fasting glucose (I-IFG), and 15 had isolated-impaired glucose tolerance (I-IGT), 5 had IFG and IGT, 14 had new diagnosed diabetes mellitus.

The study was approved by the human research ethics committee of Zhongshan hospital, Fudan University and informed consent was obtained from all subjects.

### Anthropometric and biochemical measurements

Body mass index (BMI) was calculated as body weight in kilograms divided by the square of height in meters. Waist circumference was measured at the midpoint between the inferior costal margin and the superior border of the iliac crest on the mid axillary line.

Blood samples were drawn after an overnight fast and immediately centrifuged. Serum glucose was measured by the glucoseoxidase method. Serum total cholesterol (TC), triglycerides (TG), high-density lipoprotein cholesterol (HDL-c), low-density lipoprotein cholesterol (LDL-c), and liver enzyme levels were determined by enzymatic methods with a Hitachi 7600 analyzer (Hitachi, Ltd. Tokyo, Japan).

Serum insulin (mU/L) was measured with an insulin radioimmunoassay kit (kit from Beijing North Biotechnology Research Institute), which had a reactivity of less than 0.2% to human proinsulin. The inter-assay and intra-assay coefficients of variation were 5.8% and 11.0% for insulin.

Insulin resistance (IR) and β cell function were estimated using the homeostasis model of assessment (HOMA). HOMA-IR was calculated as fasting glucose (mmol/L) × fasting insulin (mU/L)/22.5 and HOMA-β was calculated as 20 × fasting insulin (mU/L)/[fasting glucose (mmol/L)-3.5)].

Serum RBP4 (μg/ml) was measured in duplicate by a sandwich ELISA developed in-house, using affinity-chromatography purified polyclonal and monoclonal antibodies generated against recombinant human RBP4 protein. The assay system was subsequently cross-validated by Western blot analysis. The intra-assay coefficient of variation was 1.8–7.6% and inter-assay was 3.7–8.85% [30].

### Hyperglycemic clamp

A total of 17 NGT subjects (men = 6, women = 11) underwent hyperglycemic clamp. None of the subjects was taking any medications known to affect glucose and lipid metabolism. Subjects fasted 12 h before the experiments, in the experiments morning, a dorsal hand vein was cannulated in a retrograde fashion and kept in a constant-temperature device at 50-55°C for sampling arterialized venous blood. An antecubital vein was cannulated for infusion of 20% glucose. After a 30-min baseline period, serum glucose concentrations were increased to 13 mmol/L and subsequently maintained at this level for 150 min using the glucose clamp technique. During the experiments, blood samples for serum insulin determinations were obtained at -30,-15, 0, 2, 4, 6, 8, 10, 20, 30, 40, 50, 60, 70, 80, 90, 100, 110, 120, 130, 140, and 150 min [31].

### Calculations

First-phase insulin secretion (1PH) was considered the sum of the serum insulin concentration from 2–10 min of the hyperglycemic clamp. Increment of 1PH was calculated as 1PH subtracting basal insulin level. Second-phase insulin secretion (2PH) was calculated as the average serum insulin concentration from 20–150 min of the clamp. Increment of 2PH was calculated as 2PH subtracting basal insulin level. The insulin sensitivity index (ISI) was determined as the average glucose metabolized rate during the last 30 min of the hyperglycemic clamp divided by the average serum insulin concentration during the same interval.

### Determination of hepatic fat content with calibrated CT

CT scans was performed using a Marconi MX8000 machine Philips by the same one experienced doctor to measure hepatic fat content of patients. CT scan was performed at a voltage of 120 kv, electricity of 292 mA, and a slice of 6.5 mm with scan time of 3 seconds. Four slices were acquired in each patient at the following levels: dome of the liver, cephalad and caudal to the hepatic hilum, and the inferior margin of the right lobe. No contrast medium was used. The average of the four Hounsfield values obtained for each slice was measured.

Test tubes containing solutions with CT scan density (CTD) similar to normal liver (“liver-equivalent”) or “fat-equivalent material” in variable proportions were prepared to mimick different degrees of hepatic fat content. At the time of the CT measurement, the test tubes were placed on the abdomen of the patient, the CT numbers of the calibrating solutions were measured at the beginning of the exam, concurrently with those of the liver. A linear regression of the relative fat content of the test tubes solution was then calculated, and the CT number of the liver of the patient derived according to the regression line. The hepatic fat content (HFC) was expressed as a percentage of the entire liver [32].

### Statistical analyses

All analyses were performed using Statistical Package for the Social Sciences version 16.0 (SPSS Inc., Chicago, Illinois, USA). Normally distributed data were expressed as mean **±** SD. Data that were not normally distributed, as determined using the Kolmogorox-Smirnov test, were logarithmically transformed before analysis. The Student’s unpaired *t* test was used for comparison between two groups. Pearson’s correlation was used to analyze the relation of serum RBP4 and other parameters. Multiple stepwise regression analysis was used to examine the association of HOMA-β and serum RBP4 and other parameters. In all statistical tests, *P* values less than 0.05 were considered significant.

## Abbreviations

RBP4: Retinol binding protein 4; NAFLD: Non-alcoholic fatty liver disease; FBG: Fasting blood glucose; 1PH: Firstphase insulin secretion; 2PH: Second-phase insulin secretion; WHR: Waist-to-hip ratio; SBP: Systolic blood pressure; DBP: Diastolic blood pressure; HFC: Hepatic fat content; ISI: Insulin sensitivity index; OGTT: Oral glucose tolerance test; NGR: Normal glucose regulation; I-IFG: Isolated-impaired fasting glucose; I-IGT: Isolated-impairedglucose tolerance; BMI: Body mass index; TC: Total cholesterol; TG: Triglycerides; HDL-c: High-density lipoprotein cholesterol; LDL-c: Low-density lipoprotein cholesterol; IR: Insulin resistance; ALT: Alanine aminotransferase; AST: Aspartate aminotransferase; ALP: Alkaline phosphatase; γ-GT: γ-glutamyltranspeptidase; FINS: Fasting insulin; 2hINS: 2 hour insulin; AIRg: Acute insulin response; AUC-I: Area under the curve for insulin.

## Competing interests

The authors declare that they have no competing interests.

## Authors’ contributions

XG designed the research and revised the manuscript; HY and XC carried out the experiment and wrote the paper; MX and HB collected samples and took charge of detection of serum RBP4; LZ and HL participated in parts of data analysis; GC and MZ were responsible for CT detection; All authors have read and approved the final version of the manuscript.

## References

[B1] Yang Q Graham TE Mody N Preitner F Peroni OD Zabolotny JM Kotani K Quadro L Kahn BB Serum retinol binding protein 4 contributes to insulin resistance in obesity and type 2 diabetes Nature 2005 436 356 362 10.1038/nature03711 16034410

[B2] Cho YM Youn BS Lee H Lee N Min SS Kwak SH Lee HK Park KS Plasma retinol-binding protein-4 concentrations are elevated in human subjects with impaired glucose tolerance and type 2 diabetes Diabetes Care 2006 29 2457 2461 10.2337/dc06-0360 17065684

[B3] Meisinger C Ruckert IM Rathmann W Doring A Thorand B Huth C Kowall B Koenig W Retinol-binding protein 4 is associated with prediabetes in adults from the general population: the Cooperative Health Research in the Region of Augsburg (KORA) F4 Study Diabetes Care 2011 34 1648 1650 10.2337/dc11-0118 21617096 PMC3120167

[B4] Xu M Li XY Wang JG Wang XJ Huang Y Cheng Q Huang HE Li R Xiang J Tan JR Retinol-binding protein 4 is associated with impaired glucose regulation and microalbuminuria in a Chinese population Diabetologia 2009 52 1511 1519 10.1007/s00125-009-1386-8 19506831

[B5] Gavi S Stuart LM Kelly P Melendez MM Mynarcik DC Gelato MC McNurlan MA Retinol-binding protein 4 is associated with insulin resistance and body fat distribution in nonobese subjects without type 2 diabetes J Clin Endocrinol Metab 2007 92 1886 1890 10.1210/jc.2006-1815 17299074

[B6] Yang Q Eskurza I Kiernan UA Phillips DA Blueher M Graham TE Kahn BB Quantitative measurement of full-length and C-terminal Proteolyzed RBP4 in serum of normal and insulin-resistant humans using a novel mass spectrometry immunoassay Endocrinology 2012 153 1519 1527 10.1210/en.2011-1750 22253430 PMC3281532

[B7] Weiping L Qingfeng C Shikun M Xiurong L Hua Q Xiaoshu B Suhua Z Qifu L Elevated serum RBP4 is associated with insulin resistance in women with polycystic ovary syndrome Endocrine 2006 30 283 287 10.1007/s12020-006-0006-3 17526940

[B8] Kowalska I Straczkowski M Adamska A Nikolajuk A Karczewska-Kupczewska M Otziomek E Gorska M Serum retinol binding protein 4 is related to insulin resistance and nonoxidative glucose metabolism in lean and obese women with normal glucose tolerance J Clin Endocrinol Metab 2008 93 2786 2789 10.1210/jc.2008-0077 18430770

[B9] Wallenius V Elias E Bergstrom GM Zetterberg H Behre CJ The lipocalins retinol-binding protein-4, lipocalin-2 and lipocalin-type prostaglandin D2-synthase correlate with markers of inflammatory activity, alcohol intake and blood lipids, but not with insulin sensitivity in metabolically healthy 58-year-old Swedish men Exp Clin Endocrinol Diabetes 2011 119 75 80 10.1055/s-0030-1265212 21104585

[B10] Chavez AO Coletta DK Kamath S Cromack DT Monroy A Folli F DeFronzo RA Tripathy D 2 Retinol-binding protein 4 is associated with impaired glucose tolerance but not with whole body or hepatic insulin resistance in Mexican Americans Am J Physiol Endocrinol Metab 2009 296 E758 E764 10.1152/ajpendo.90737.2008 19190263

[B11] Kotnik P Fischer-Posovszky P Wabitsch M RBP4: a controversial adipokine Eur J Endocrinol 2011 165 703 711 10.1530/EJE-11-0431 21835764

[B12] Hongmei Y Xin G Liu M Qian GU Jian GAO Changes of β-cell function and insulin sensitivity in subjects winl non-alcoholic fatty liver disease and normal glucose regulation Chin J Endocrinol Metab 2007 1 12 15

[B13] Bian H Lin H Rao S Yao X Zeng M Zhou J Weiping J Gao X Association of liver fat content with insulin resistance and islet βcell function in individuals with varions statuses of glucose metabolism Chin J Endocrinol Metab 2010 26 535 540

[B14] Seo JA Kim NH Park SY Kim HY Ryu OH Lee KW Lee J Kim DL Choi KM Baik SH 1 Serum retinol-binding protein 4 levels are elevated in non-alcoholic fatty liver disease Clin Endocrinol (Oxf) 2008 68 555 560 10.1111/j.1365-2265.2007.03072.x 17941908 PMC2344088

[B15] Broch M Vendrell J Ricart W Richart C Fernandez-Real JM Circulating retinol-binding protein-4, insulin sensitivity, insulin secretion, and insulin disposition index in obese and nonobese subjects Diabetes Care 2007 30 1802 1806 10.2337/dc06-2034 17416795

[B16] Li L Wang C Bao Y Wu H Lu J Xiang K Jia W Serum retinol-binding protein 4 is associated with insulin secretion in Chinese people with normal glucose tolerance J Diabetes 2009 1 125 130 10.1111/j.1753-0407.2009.00024.x 20929509

[B17] Stefan N Hennige AM Staiger H Schleicher E Fritsche A Haring HU Circulating retinol-binding protein-4, insulin sensitivity, insulin secretion, and insulin disposition index in obese and nonobese subjects: response to Broch et al Diabetes Care 2007 30 e91 author reply e92 10.2337/dc07-0767 17855276

[B18] Blaner WS STRA6, a cell-surface receptor for retinol-binding protein: the plot thickens Cell Metab 2007 5 164 166 10.1016/j.cmet.2007.02.006 17339024

[B19] Chertow BS Effects of vitamin a deficiency and repletion on Rat insulin secretion in vivo and in vitro from isolated islets J Clin Invest 1987 79 163 169 10.1172/JCI112778 3025258 PMC424013

[B20] Refai E Dekki N Yang SN Imreh G Cabrera O Yu L Yang G Norgren S Rossner SM Inverardi L Transthyretin constitutes a functional component in pancreatic beta-cell stimulus-secretion coupling Proc Natl Acad Sci U S A 2005 102 17020 17025 10.1073/pnas.0503219102 16286652 PMC1287967

[B21] Sivaprasadarao AFJ The interaction of retinol-binding protein with its plasma-membrane receptor Biochem J 1988 255 561 569 2849420 PMC1135265

[B22] Zanotti G Berni R Plasma retinol-binding protein: structure and interactions with retinol, retinoids, and transthyretin Vitam Horm 2004 69 271 295 15196886 10.1016/S0083-6729(04)69010-8

[B23] Graham TE Wason CJ Bluher M Kahn BB Shortcomings in methodology complicate measurements of serum retinol binding protein (RBP4) in insulin-resistant human subjects Diabetologia 2007 50 814 823 10.1007/s00125-006-0557-0 17294166

[B24] Lee DC Lee JW Im JA Association of serum retinol binding protein 4 and insulin resistance in apparently healthy adolescents Metabolism 2007 56 327 331 10.1016/j.metabol.2006.10.011 17292720

[B25] Balagopal P Graham TE Kahn BB Altomare A Funanage V George D Reduction of elevated serum retinol binding protein in obese children by lifestyle intervention: association with subclinical inflammation J Clin Endocrinol Metab 2007 92 1971 1974 10.1210/jc.2006-2712 17341558

[B26] An C Wang H Liu X Li Y Su Y Gao X Jiang W Serum retinol-binding protein 4 is elevated and positively associated with insulin resistance in postmenopausal women Endocr J 2009 56 987 996 10.1507/endocrj.K09E-096 19671994

[B27] Reinehr T Stoffel-Wagner B Roth CL Retinol-binding protein 4 and its relation to insulin resistance in obese children before and after weight loss J Clin Endocrinol Metab 2008 93 2287 2293 10.1210/jc.2007-2745 18397979 PMC2729181

[B28] McInnes KJ Smith LB Hunger NI Saunders PTK Andrew R Walker BR Deletion of the androgen receptor in adipose tissue in male mice elevates retinol binding protein 4 and reveals independent effects on visceral Fat mass and on glucose homeostasis Diabetes 2012 61 1072 1081 10.2337/db11-1136 22415878 PMC3331763

[B29] Palomar-Morales MMS Mendoza-Rodríguez CA Cerbón MA The protective effect of testosterone on streptozotocin-induced apoptosis in beta cells is sex specific Pancreas 2010 39 193 200 10.1097/MPA.0b013e3181c156d9 20093993

[B30] Qi Q Yu Z Ye X Zhao F Huang P Hu FB Franco OH Wang J Li H Liu Y Lin X 1 Elevated retinol-binding protein 4 levels are associated with metabolic syndrome in Chinese people J Clin Endocrinol Metab 2007 92 4827 4834 10.1210/jc.2007-1219 17878249

[B31] Li H Bao Y Xu A Pan X Lu J Wu H Lu H Xiang K Jia W Serum fibroblast growth factor 21 is associated with adverse lipid profiles and gamma-glutamyltransferase but not insulin sensitivity in Chinese subjects J Clin Endocrinol Metab 2009 94 2151 2156 10.1210/jc.2008-2331 19318452

[B32] Ricci CLR Gioulis E Bosco M Pollesello P Masutti F Crocè LS Paoletti S de Bernard B Tiribelli C Dalla Palma L Noninvasive in vivo quantitative assessment of fat content in human liver J Hepatol 1997 27 108 113 10.1016/S0168-8278(97)80288-7 9252082

